# Late histological findings in symptomatic COVID-19 patients: A case report

**DOI:** 10.1097/MD.0000000000021046

**Published:** 2020-07-10

**Authors:** Alberto Aiolfi, Barbara Bruni, Tullio Biraghi, Andrea Montisci, Antonio Miceli, Barbara Baronio, Desmond Khor, Silvia Cirri, Francesco Donatelli, Claudio Clemente, Davide Bona

**Affiliations:** a Department of Biomedical Science for Health, Division of General Surgery, Istitituto Clinico Sant’Ambrogio, University of Milan; b Department of Pathology, I.R.C.C.S. Policlinico San Donato, Milano; c Department of Anaesthesia and Intensive Care, Cardiothoracic Center, Istituto Clinico Sant’Ambrogio, University of Milan; d Chair of Cardiac Surgery, Department of Cardiothoracic Center, Istituto Clinico Sant’Ambrogio; University of Milan, Milano, Italy.

**Keywords:** COVID-19 pneumonia, novel coronavirus, pathology

## Abstract

**Rationale:**

Although there have been several studies describing clinical and radiographic features about the novel coronavirus (COVID-19) infection, there is a lack of pathologic data conducted on biopsies or autopsies.

**Patient concerns:**

A 56-year-old and a 70-year-old men with fever, cough, and respiratory fatigue were admitted to the intensive care unit and intubated for respiratory distress.

**Diagnosis:**

The nasopharyngeal swab was positive for COVID-19 and the chest Computed Tomography (CT) scan showed the presence of peripheral and bilateral ground-glass opacities.

**Interventions:**

Both patients developed pneumothoraces after intubation and was managed with chest tube. Due to persistent air leak, thoracoscopies with blebs resection and pleurectomies were performed on 23rd and 16th days from symptoms onset.

**Outcomes:**

The procedures were successful with no evidence of postoperative air-leak, with respiratory improvement. Pathological specimens were analyzed with evidence of diffuse alveolar septum disruption, interstitium thickness, and infiltration of inflammatory cells with diffuse endothelial dysfunction and hemorrhagic thrombosis.

**Lessons:**

Despite well-known pulmonary damages induced by the COVID-19, the late-phase histological changes include diffused peripheral vessels endothelial hyperplasia, in toto muscular wall thickening, and intravascular hemorrhagic thrombosis.

## 1 Introduction

The outbreak of the novel coronavirus (COVID-19) infection was first reported in Wuhan, China. The subsequent spread of the disease led to the worldwide diffusion since the World Health Organization declared the pandemic state. Although there have been several studies describing the clinical and radiographic features about the COVID-19 infection,^[1,2]^ there is a lack of pathologic informations conducted on biopsies or autopsies.^[3–5]^ A previous study reported data of 2 asymptomatic patients treated for lung adenocarcinoma and retrospectively diagnosed with COVID-19.^[3]^ The described pathological features were accidental and likely to be early in asymptomatic patients. Therefore, the histological findings associated with advanced phase of the disease remain unclear. With this report we described the late stage histological findings of 2 symptomatic COVID-19 patients intubated for respiratory insufficiency.

## 2 Case presentation

Written informed consent was obtained from the patients relatives for publication of the case details and accompanying images.

### 2.1 Case #1

**Figure 1:**
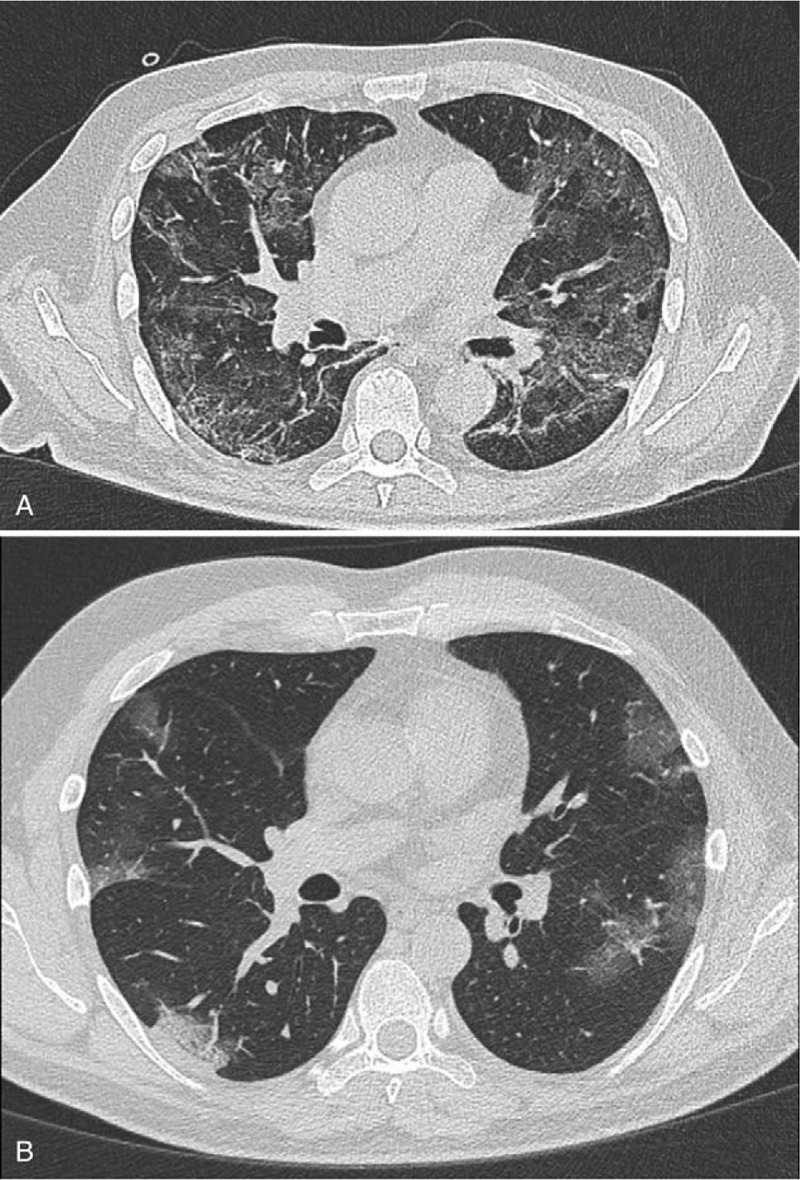
A-B. Chest Computed Tomography scan showing bilateral ground glass opacities with multilobar involvement.

**Figure 2:**
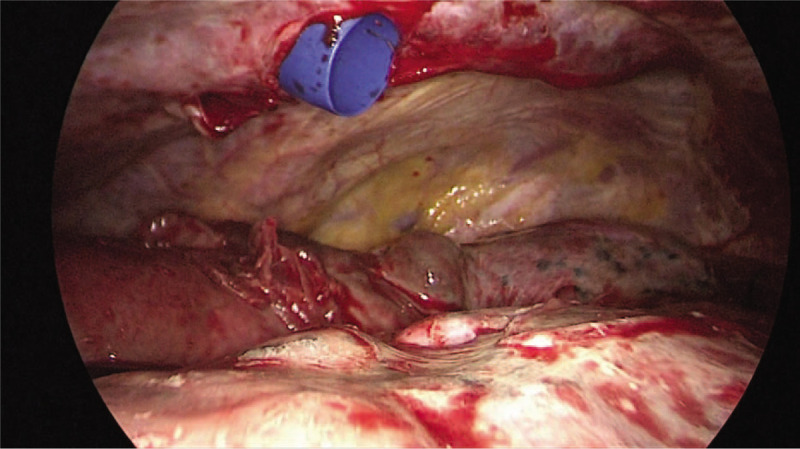
The thoracoscopic evaluation showed a stiff, edematous, and congestive lung parenchyma with superficial blebs.

A 56-year old man, active smoker, was admitted to our hospital with fever, cough, and respiratory distress. Admission laboratory exams were: WBC: 18.200/mm^3^; neutrophils: 54%; CRP: 21.3 mg/dl, D-dimer: 15.442 μg/L. The nasopharyngeal swab was positive for COVID-19 and the chest Computed Tomography (CT) scan showed presence of peripheral and bilateral ground-glass opacities (Fig. 1A). He was admitted to the Intensive Care Unit (ICU) and later intubated for respiratory insufficiency. Ventilator settings were: tidal volume 6 ml/kg and PEEP 10 cmH_2_O. The patient developed post-intubation pneumothorax and was managed with a 28 Fr chest tube. Due to persistent air leak, thoracoscopy (Fig. 2) with blebs resection and pleurectomy was performed on day 23rd after symptoms onset.^[6]^

### 2.2 Cases #2

A 70-year old man without comorbidities was admitted to our hospital with fever, fatigue, and respiratory distress. Laboratory exams were: WBC: 14.700/mm^3^; neutrophils: 48%; CRP: 16.1 mg/dl, D-dimer: 12.891 μg/L. Diagnosis of COVID-19 was done with positive nasopharyngeal swab and evidence of bilateral ground-glass opacities was shown on thorax CT scan (Fig. 1B). He was admitted to the ICU and intubated for respiratory insufficiency. Ventilator settings were: tidal volume 6 ml/kg and PEEP 8 cmH_2_O. He also developed pneumothorax required the positioning of a 28 Fr chest tube. Again, because of persistent air leak, thoracoscopy with blebs resection and pleurectomy was performed on 16th day from symptoms onset.^[6]^

During the hospital course, both patients received vitamin C, hydroxychloroquine, and azithromycin. The surgical procedures were successful in both patients with no evidence of persistent air-leak and respiratory improvement. Drains were removed in both patients on postoperative day 2. Despite the initial respiratory improvement patient#1 passed away 56 days after symptoms onset because of cardiac failure. Patient #2 was estubated and discharged from the ICU 28 days after symptoms onset.

**Figure 3:**
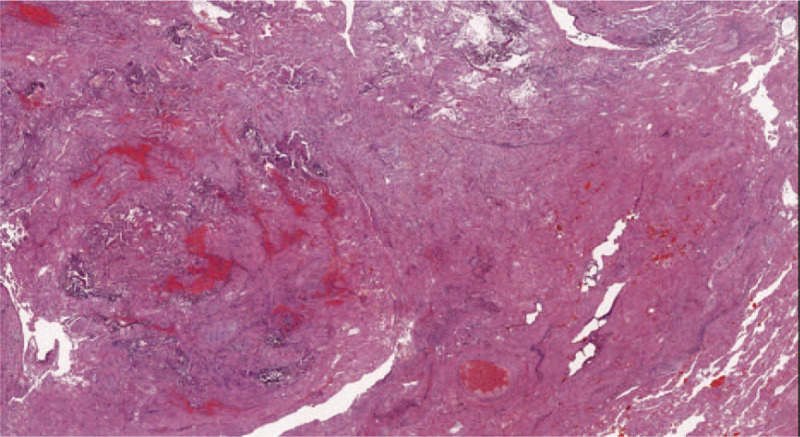
Representative hematoxylin and eosin section of the lung (15X) - Massive alveoli damage with hemorrhagic necrosis end interstitial fibrosis.

**Figure 4:**
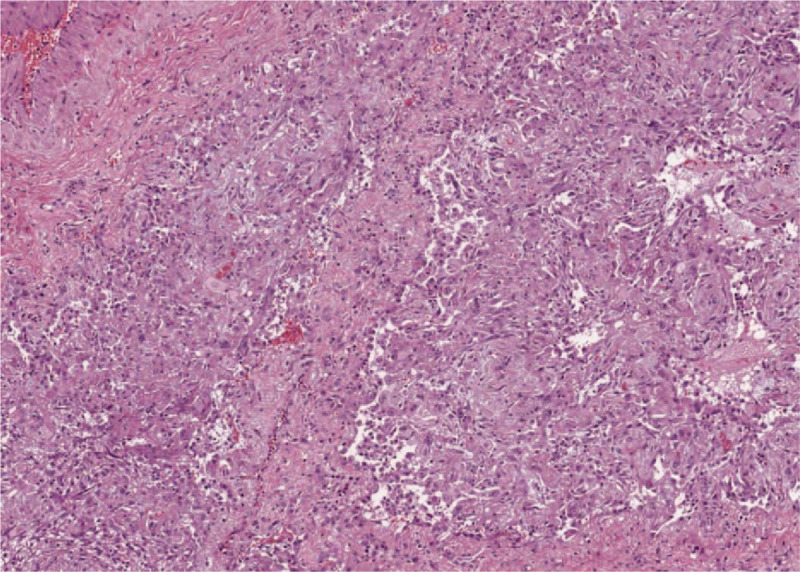
Representative hematoxylin and eosin section of the lung (45X) - alveolar damage with desquamate epithelial cells and inflammatory cells infiltration.

**Figure 5:**
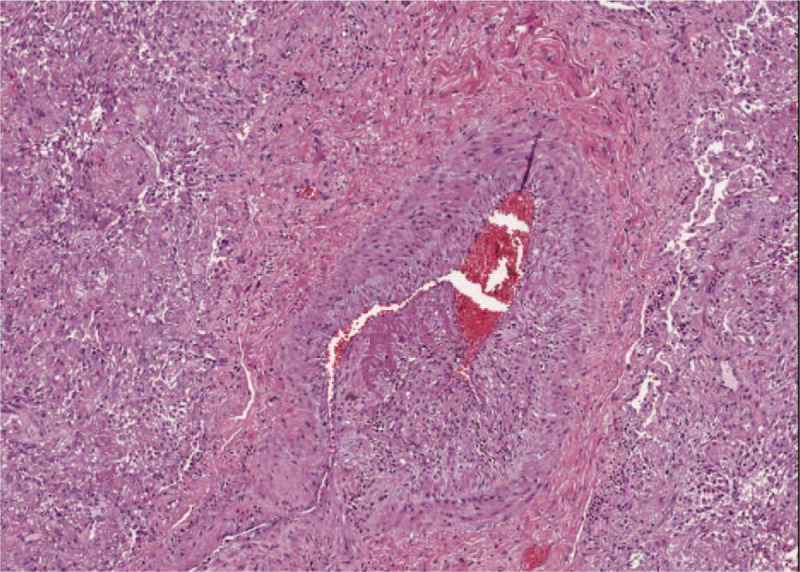
Representative hematoxylin and eosin section of the lung (45X) – Intravascular hemorrhagic thrombosis, with in toto muscular wall thickening and endothelial hyperplasia.

Specimens were analyzed and pathological findings showed no evidence of pathognomonic lesions. The gross tissue evaluation showed diffuse congestive appearance with edema and haemorrhagic necrosis. The main pathological changes consisted is alveolar damage with septa disruption, desquamation, edema, and proteinaceous exudates (Fig. 3). Alveolar congestion was prominent and contained edema fluid, desquamated epithelial and inflammatory cells. The interstitial tissue was mildly fibrotic and thickened with cells infiltration (monocytes, lymphocytes, plasma cells, and multinucleate giant cells) (Fig. 4). Immunohistochemistry showed positive results for TTF-1, NapsineA, CD3, CD34, CD5, CD8, CD31, CK7, and Collagen Type IV. Peripheral vessels showed diffused endothelial hyperplasia, in toto muscular wall thickening, and massive intravascular hemorrhagic thrombosis (Fig. 5).

## 3 Discussion

The novel coronavirus 2019 (COVID-19), also known as severe acute respiratory syndrome coronavirus 2 (SARS-CoV-2) is an enveloped, non-segmented positive-sense RNA virus causing severe bilateral pneumonia and acute respiratory distress syndrome (ARDS) in patients. The most commonly reported symptoms are fever, cough, myalgia, and dyspnea.^[7]^ Up to 15% of patients may develop severe symptoms and respiratory fatigue requiring mechanical ventilation and intensive care unit management.^[8]^ Diagnosis is made with a nasopharyngeal swab, immunoglobulin serum tests, and evidence of bilateral and multiple patchy or ground glass appearance at chest CT scan.

Although clinical and radiographic findings in COVID-19 patients have been largely reported, there is a lack of pathologic informations conducted on biopsies or autopsies and the literature is scarce. The early histologic changes of 2 patients that underwent lung lobectomy for adenocarcinoma and retrospectively diagnosed with COVID-19 have been previously described.^[3]^ Edema, proteinaceous exudate, focal reactive hyperplasia of pneumocytes with inflammatory cellular infiltration were prominent in the pathological specimens. To our knowledge, this report by Tian and colleagues represents the first description of pulmonary changes induced by COVID-19.^[3]^ However, the described tissue alterations were the early-phase changes in asymptomatic subjects and may not be totally accurate for symptomatic patients. Therefore, our study managed to broaden Tian and colleagues report by describing the pathological changes of 2 symptomatic COVID-19 patients intubated for respiratory distress. Bronchiolitis and alveolitis with proliferation, atrophy, and desquamation of alveolar epithelial cells were present confirming the direct tropism of the virus for alveolar cells. This is in accordance with a recently published series describing postmortem findings of firm and severely congested lung parenchyma with concomitant exudative diffuse alveolar damage.^[9]^ The selective tropism of the COVID-19 for the angiotensin converting enzyme 2 (ACE2), exposed in type II alveolar cells, results in cells damage and apoptosis with consequent acute alveoli injury, septum disruption, and pulmonary edema. The interstitial tissue is thickened due to sustained fibroblast activation with a significant amount of infiltrated inflammatory cells triggered via the circulating pro-inflammatory “cytokine storm” (i.e. TNF, IL-1β, IL-6, etc.).^[10]^ The thickened interstitium correspond to the characteristic radiographic findings of peripheral ground-glass opacity detected by chest computed tomography. Diffuse peripheral vessels involvement, endothelial thickening, and diffuse intraluminal hemorrhagic thrombosis, consistent with a generalized microvascular injury was found in both cases. ACE2 receptors are expressed by endothelial cells and recent findings showed direct evidence of viral elements within endothelial cytoplasm.^[11]^ It is likely that the direct viral and complement-mediated endothelial injury cause a widespread alteration of the endothelial homeostasis with consequent microvascular dysfunction and shift of the vascular equilibrium towards a procoagulant state.^[12]^ This further support the recently described findings of capillarostasis, vascular congestion, alveolar capillaries microthrombi, and pulmonary embolism.^[13,14]^ These features may correlate with the late phase of disease-induced coagulopathy and consequent increase of lung dead space, presumably cause of respiratory distress necessitating mechanical support.

## 4 Conclusion

To our knowledge, this is the first report describing the late phase histological findings of 2 symptomatic COVID-19 patients intubated for respiratory distress. Despite the well-known pulmonary damage, the late-phase tissue alterations include diffused peripheral vessels endothelial hyperplasia, in toto muscular wall thickening, and intravascular hemorrhagic thrombosis. These findings may provide new insights into the specific COVID-19 pneumonia pathogenesis, which might be useful for focused and timely therapeutic strategies.

## Author contributions

Study conception and design: Aiolfi, Biraghi, Bona. Acquisition of data: Aiolfi and Bona. Analysis and interpretation of data: Aiolfi, Bruni, Clemente, Donatelli. Drafting of manuscript: Aiolfi and Khor. Critical revision of manuscript: Aiolfi, Cirri, Miceli, Montisci, Donatelli, Bona.

## References

[1] Zhu N Zhang D Wang W A novel coronavirus from patients with pneumonia in China, 2019. N Engl J Med 2020;doi.org/10.1056/NEJMoa2001017. 10.1056/NEJMoa2001017 PMC7092803 31978945

[2] Chung M Bernheim A Mei X CT imaging features of 2019 novel coronavirus (2019-nCoV). Radiology 2020;295:202–7. 32017661 10.1148/radiol.2020200230 PMC7194022

[3] Tian S Hu W Niu L Pulmonary pathology of early-phase 2019 novel coronavirus (COVID-19) pneumonia in two patients with lung cancer. J Thorac Oncol 2020;pii: S1556-0864(20)30132-5. doi: 10.1016/j.jtho.2020.02.010. [Epub ahead of print]. 10.1016/j.jtho.2020.02.010 PMC7128866 32114094

[4] Xu Z Shi L Wang Y Pathological findings of COVID-19 associated with acute respiratory distress syndrome. Lancet Respir Med 2020;8:420–2. Epub 2020 Feb 18. 32085846 10.1016/S2213-2600(20)30076-X PMC7164771

[5] Luo W Yu H Gou Clinical pathology of critical patient with novel coronavirus pneumonia (COVID-19). Preprints 2020;2020020407.

[6] Aiolfi A Biraghi T Montisci A Management of persistent pneumothorax with thoracoscopy and blebs resection in covid-19 patients. Ann Thorac Surg 2020;pii: S0003-4975(20)30604-4. 10.1016/j.athoracsur.2020.04.011 PMC7185014 32353441

[7] Wan S Xiang Y Fang W Clinical features and treatment of COVID-19 patients in northeast Chongqing. J Med Virol 2020;1–0. doi:10.1002/jmv.25783. 32198776 10.1002/jmv.25783 PMC7228368

[8] Wang Wang D Hu B Hu C Clinical characteristics of 138 hospitalized patients with 2019 novel coronavirus-infected pneumonia in Wuhan, China. JAMA 2020;doi.org/10.1001/jama.2020.1585. 10.1001/jama.2020.1585 PMC7042881 32031570

[9] Menter T Haslbauer JD Nienhold R Post-mortem xxamination of COVID19 patients reveals diffuse alveolar damage with severe capillary congestion and variegated findings of lungs and other organs suggesting vascular dysfunction. Histopathology 2020;doi: 10.1111/his.14134. Online ahead of print. 10.1111/his.14134 PMC7496150 32364264

[10] Mehta P McAuley DF Brown M HLH across speciality collaboration, UK. COVID-19: consider cytokine storm syndromes and immunosuppression. Lancet 2020;395:1033–4. Epub 2020 Mar 16. 32192578 10.1016/S0140-6736(20)30628-0 PMC7270045

[11] Varga Z Flammer AJ Steiger P Endothelial cell infection and endotheliitis in COVID-19. Lancet 2020;pii: S0140-6736(20)30937-5. doi: 10.1016/S0140-6736(20)30937-5. [Epub ahead of print]. 10.1016/S0140-6736(20)30937-5 PMC7172722 32325026

[12] Bikdeli B Madhavan MV Jimenez D COVID-19 and thrombotic or thromboembolic disease: implications for prevention, antithrombotic therapy, and follow-up. J Am Coll Cardiol 2020;[PMID: 32311448] doi:10.1016/j.jacc.2020.04.031. 10.1016/j.jacc.2020.04.031 PMC7164881 32311448

[13] Wichmann D Sperhake JP Lütgehetmann M Autopsy findings and venous thromboembolism in patients with COVID-19. Ann Intern Med 2020;M20–2003. doi: 10.7326/M20-2003. 10.7326/L20-1206 33316197

[14] Lax SF Skok K Zechner P Pulmonary arterial thrombosis in COVID-19 with fatal outcome: results from a prospective, single-center, clinicopathologic case series. Ann Intern Med 2020;doi: 10.7326/M20-2566. Online ahead of print. 10.7326/M20-2566 PMC7249507 32422076

